# Concurrent Tuberculosis and COVID-19 Testing from a Single Sputum Specimen for Enhanced Disease Detection

**DOI:** 10.3390/diagnostics15060720

**Published:** 2025-03-13

**Authors:** Anura David, Leisha Genade, Lesley Erica Scott, Manuel Pedro da Silva, Lyndel Singh, Wendy Stevens, Neil Martinson

**Affiliations:** 1 Wits Diagnostic Innovation Hub, Health Sciences Research Office, Faculty of Health Sciences, University of the Witwatersrand, Johannesburg 2193, South Africa; lesley.scott@wits.ac.za (L.E.S.); lyndel.singh@witsdih.ac.za (L.S.); wendy.stevens@wits.ac.za (W.S.); 2 Perinatal HIV Research Unit (PHRU), University of the Witwatersrand, Johannesburg 2193, South Africa; genadel@phru.co.za (L.G.); martinson@phru.co.za (N.M.); 3 National Priority Program, National Health Laboratory Service, Johannesburg 2193, South Africa; pedro.dasilva@nhls.ac.za; 4 Center for TB Research, Johns Hopkins University, Baltimore, MD 21218, USA

**Keywords:** *Mycobacterium tuberculosis*, bi-disease testing, COVID-19, tuberculosis, SARS-CoV-2

## Abstract

**Background/Objectives:** Tuberculosis (TB) and SARS-CoV-2 share similar symptoms and transmission routes. In early 2021, USAID and Stop TB Partnership recommended an integrated approach for simultaneous COVID-19 and TB testing in high TB burden countries for individuals with respiratory symptoms. In this evaluation, we tested a single sputum for both SARS-CoV-2 and *Mycobacterium tuberculosis* complex (MTBC) from participants at two healthcare facilities in South Africa. The diagnostic accuracy of the Xpert Xpress SARS-CoV-2 (Xpress) assay using a sputum swab capture method was assessed by comparing the results with routine SARS-CoV-2 testing, while also determining the prevalence of TB and TB-COVID-19 co-infection in the study population. **Methods:** A total of 2274 individuals were screened for enrolment. Eligibility included the presence of respiratory symptoms, close contact with a person with TB, TB diagnosis in the last two years or a person living with HIV. Sputum from 1032 participants was tested on the Xpress assay using a swab capture method while residual sputum was tested on the Xpert MTB/RIF Ultra assay for MTBC and rifampicin-resistance detection. Concordance between the Xpress assay and routine SARS-CoV-2 testing was assessed. **Results:** The Xpress assay detected SARS-CoV-2 in 183/1032 (18%) participants, TB was detected in 35/1032 (3%) participants and 10/1032 (1%) participants were co-infected with TB and COVID-19. The Xpress assay showed substantial agreement with routine testing (Kappa: 0.755). **Conclusions:** The study findings underscore a substantial identification of TB and rifampicin-resistant TB that would have been missed if bi-disease testing was not performed. In addition, the sputum swab capture method demonstrated reliable performance for SARS-CoV-2 detection.

## 1. Introduction

Since the onset of the COVID-19 pandemic, TB and COVID-19 have been among the leading causes of death from a single infectious agent [1], with TB reclaiming the top position in 2023 [2]. Health service disruption during the COVID-19 pandemic impacted the care of people with TB and caused a global decline in TB testing and diagnosis [1,3,4].

TB recovery plans encourage active case finding and aim to address the impact of COVID-19 by finding “missing” TB patients, ensuring linkage to care for those diagnosed, and strengthening retention into care and TB prevention. For South Africa (SA), the TB Recovery plan [5,6] includes Targeted Universal Testing for TB (TUTT), which tests individuals in three high-risk groups including contacts of individuals with active disease, individuals with recent TB diagnosis (last two years) and people living with HIV (PLHIV); this was trialed immediately before the onset of the COVID-19 pandemic in SA.

Previously, the diagnosis of TB, in SA, relied on the passive, self-presentation of individuals with symptoms at health care facilities (HCFs) [7]. The WHO-recommended four-symptom screen (W4SS) for TB [8] was primarily used to identify individuals for further investigation—in most instances, this would be sputum collection, subjected to molecular WHO-approved rapid diagnostic tests (mWRDs) for TB [8]. The problem with this approach is that up to half of all TB cases are asymptomatic, potentially contributing to transmission [9]. Active, community or facility-based interventions such as the TUTT approach are, therefore, crucial in identifying additional TB cases. However, their implementation was impacted during COVID-19 due to restrictions on movement, the reduced collection of sputum and symptoms common to both TB and COVID-19, making it difficult to distinguish pathogens without testing [10,11]. Typically, upper respiratory specimens such as nasopharyngeal (NP) swabs are used for SARS-CoV-2 detection, whereas for TB, sputum is required for molecular testing and sputum culture remains the gold standard for TB diagnosis [12]. Whilst the interaction between TB and COVID-19 is still being investigated, studies suggest that TB status and history might play a role in the development and progression of COVID-19 infection [13,14]. Furthermore, TB disease is reported to be more common among patients with SARS-CoV-2 infection than in those with bacterial or other viral infections [11]. SARS-CoV-2 infection has also been shown to mask or intensify TB infection with potential severe consequences [15].

In early 2021, to minimize and reverse the impact of COVID-19 on TB diagnosis, USAID and Stop TB Partnership recommended simultaneous testing for COVID-19 and TB in high TB burden countries, using a NP swab and sputum for individuals presenting with respiratory symptoms [16]. Simultaneous rapid molecular testing for TB and SARS-CoV-2 has the advantage of reducing the transmission of both pathogens in the community and HCFs [17]. This approach was piloted at five HCFs in the Greater Accra Region in Ghana and demonstrated the potential of improving case detection for both COVID-19 and TB [18].

We previously conducted a small pilot study demonstrating the feasibility of detecting both SARS-CoV-2 and *Mycobacterium tuberculosis* complex (MTBC) from a single sputum sample [19]. To build on these findings, this larger study aimed to validate the approach in a broader population, including both symptomatic and asymptomatic individuals at primary healthcare clinics (PHCs) who would have otherwise only undergone routine SARS-CoV-2 testing. We assessed the performance of the Xpert Xpress SARS-CoV-2 (Xpress) assay (Cepheid, Sunnyvale, CA, USA) using a sputum swab capture method by comparing the results with routine SARS-CoV-2 testing and determining the prevalence of TB, rifampicin (RIF)-resistant TB and TB–COVID-19 co-infection in the study population.

## 2. Materials and Methods

### 2.1. Sample Size

Previous findings in SA showed that around 1.2% of patients with symptoms requiring a SARS-CoV-2 test were positive for MTBC, using Xpert MTB/RIF Ultra (Ultra) assay (Cepheid, Sunnyvale, CA, USA), about double the general TB prevalence in Gauteng. With decreasing COVID-19 cases and an assumed difference of 0.09, we estimated enrolling at least 1000 participants, yielding around 90 TB patients and at least 200 COVID-19 patients, depending on a potential fourth COVID-19 surge.

### 2.2. Participant Recruitment

In this prospective, cross-sectional study, participant recruitment occurred between November 2021 and June 2022. Adults (≥18 years) attending two large PHCHs in Soweto, Johannesburg, SA were invited to enroll in the study. Both clinics, at the beginning of recruitment, had a respiratory queue where patients with respiratory symptoms had an NP swab taken for routine testing for SARS-CoV-2. This changed over time and close to the end of the study, patients required a doctor’s confirmation for testing for SARS-CoV-2. Inclusion criteria included individuals with respiratory symptoms awaiting a public sector SARS-CoV-2 molecular test or those belonging to a high-risk-for-TB group. All participants had the same data collected, detailing their symptoms and including both their W4SS and upper respiratory and constitution symptoms (Table S1: Symptoms reported by participants). Anthropometrics and vital signs were also captured.

### 2.3. Specimen Collection

Once informed consent was obtained, one NP swab, one tongue swab (TS), ~3 mL saliva and ~4 mL sputum were collected from each participant. Sputum and saliva were collected in the open (away from other participants and patients) under the supervision of a study staffer who remained at least 2 m away from the participant and wore appropriate protective personal equipment.

### 2.4. Specimen Testing

#### 2.4.1. Nasopharyngeal Swabs

Each participant’s NP swab was sent to the routine National Health Laboratory Service, or if NP SARS-CoV-2 testing was not indicated by current policy, to the research laboratory in Braamfontein, Johannesburg for SARS-CoV-2 testing. Staff conducting the testing were blinded to the Xpress assay results. Assays used for routine testing included Xpress, cobas^®^ SARS-CoV-2 (Roche, Basel, Switzerland), Allplex™ SARS-CoV-2 (Seegene, Seoul, Republic of Korea) and TaqPath™ COVID-19 (Thermo Fisher Scientific, Waltham, MA, USA).

#### 2.4.2. Sputum

Sputum was sent to the research lab for MTBC investigation using Ultra and for SARS-CoV-2 detection using the Xpress assay, using a swab capture method, previously described [19,20]. Briefly, nylon flocked swabs were inserted into sputum and twirled around, and any material collected on the swab was re-suspended in phosphate buffer (PB). The eluate was tested for SARS-CoV-2. Xpress results were compared to routine PCR and only specimens with valid results were included in the analysis. Staff performing testing were blinded to routine PCR results. Concordance between the Xpress assay and routine PCR was assessed using percent agreement and the kappa statistic using Stata version 16.1 (StataCorp, College Station, TX, USA). Residual sputum was tested on the Ultra, as per manufacturer instructions.

#### 2.4.3. Saliva and Tongue Swabs

Saliva and tongue swabs were stored for future testing.

## 3. Results

### 3.1. Participant Characteristics

**Figure 1:**
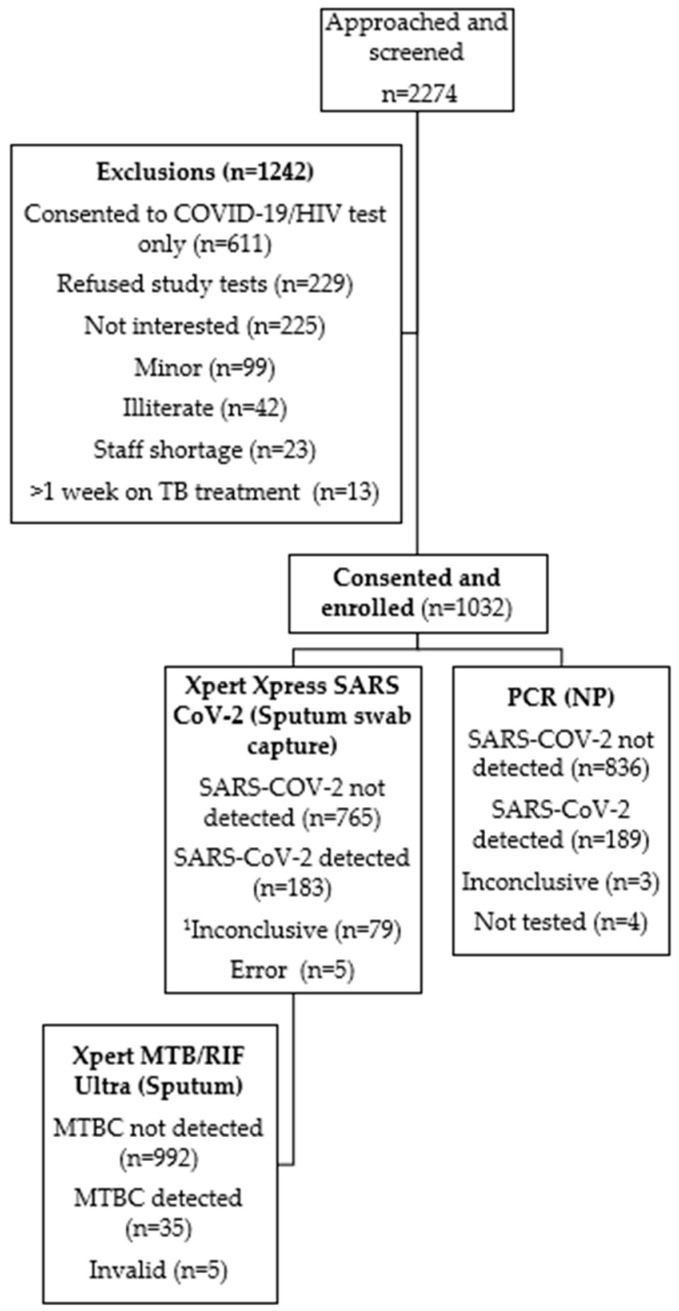
Description of participant recruitment, specimen testing and data descriptions for statistical analysis. HIV, human immunodeficiency virus; MTBC, *Mycobacterium tuberculosis* complex; NP, nasopharyngeal swab; PCR, polymerase chain reaction. Xpert Xpress SARS-CoV-2 was used to detect SARS-CoV-2 on sputum swabs, while Xpert MTB/RIF Ultra was used to detect MTBC and rifampicin resistance-associated mutations in sputum. Routine SARS-CoV-2 testing was performed on nasopharyngeal swabs. ^1^ Inconclusive result determined using National Health Laboratory Services algorithm.

A total of 2274 individuals were screened for enrolment and informed consent was obtained from 1032 participants (Figure 1). Participants’ mean age was 41 years (interquartile range, 18–84 years) (Table 1); 45% were male and 24% were PLHIV. Participant enrolment was similar between the two study sites but participants from one site had a longer duration of symptoms prior to seeking healthcare (up to 62 days). At least 90% of PLHIV recruited were receiving antiretroviral (ARV) therapy at study enrolment. Hypertension was the most common co-morbidity reported (16%) by participants across both sites. Cough was the most common symptom reported (94%). There were 15/1032 (1.5%) participants who, despite not reporting having them, had elevated random glucose levels.

### 3.2. TB and Drug-Resistant TB

A total of 35/1032 (3.4%) participants had bacteriological confirmation of TB using Ultra (Figure 1 and Table 2). Among these 35 individuals, 34 (97.1%) reported respiratory symptoms. Among the 35 participants, 16 (45.7%) were considered to be in at least one high-risk-for-TB group; three experienced a previous episode of TB, three were in contact with a person diagnosed with TB and ten were HIV-positive. Furthermore, seven participants (20%) had Rif resistance detected on Ultra. Five specimens produced unsuccessful results.

### 3.3. COVID-19 Routine PCR Results

**Figure 2:**
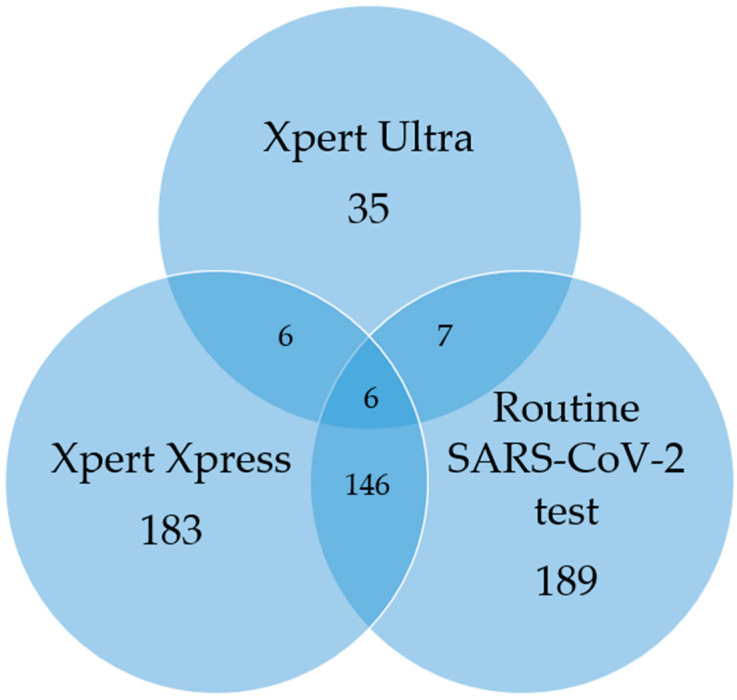
Venn diagram illustrating the number of participants who tested positive for Mycobacterium tuberculosis complex (MTBC) and/or SARS-CoV-2 using Xpert MTB/RIF Ultra, Xpert Xpress SARS-CoV-2 and routine SARS-CoV-2 PCR testing. Xpert Xpress SARS-CoV-2 was used to detect SARS-CoV-2 on sputum swabs, while Xpert MTB/RIF Ultra was used to detect MTBC and rifampicin resistance-associated mutations in sputum. Routine SARS-CoV-2 testing was performed on nasopharyngeal swabs. The numbers represent the distribution of positive results among the three testing methods. The overlapping areas indicate participants with concordant results across multiple methods.

A total of 189/1028 (18.4%) participants tested positive for SARS-CoV-2 using routine PCR (Figure 2). Of these, 8/189 (4.2%) were asymptomatic (Table 2). Symptom duration among participants ranged from 1- 38 days. Cough and headache accounted for >80% of symptoms reported. Hypertension was the most common co-morbidity reported by 33/189 (17.5%) participants. Results were unavailable for four participants due to either missing specimens or laboratory rejection. Three specimens produced inconclusive results.

### 3.4. Xpert Xpress SARS-CoV-2 Results

Of the 1032 participants, 183 (18%) tested positive for SARS-CoV-2 using the Xpress sputum swab capture method (Table 2). Cough was the leading symptom reported by 155/171 (91%) participants. The average duration of symptoms reported for this cohort is 4.7 days (range: 1–38 days). Fifteen participants reported having a previous TB episode. Five specimens produced unsuccessful results. A total of 79/1032 (8%) results were recorded as inconclusive (as per National Health Laboratory Services (NHLS) reporting where a single E or N2 gene has a Ct >38 or where both E and N2 genes have Ct >40 (internal memo Dr M.P. da Silva)). Of the 941 participants who demonstrated valid results for the Xpress assay and routine PCR, 869 (92%) (Table 3) showed substantial agreement between the two assays (Kappa: 0.755).

### 3.5. TB/SARS-CoV-2 Co-Infection

A total of 10/1032 (1%) patients had both MTBC, and SARS-CoV-2 detected, either on routine PCR or the Xpress sputum capture method (Table 2 and Figure 2). None of these ten co-infected participants were contacts of people with active TB or reported a previous TB episode. Of the ten individuals, nine (90%) had not received a COVID-19 vaccine. In addition, 5/10 (50%) were HIV-positive. All participants reported respiratory symptoms for an average duration of 9.6 days.

## 4. Discussion

In this study, bi-disease testing for COVID-19 and TB was investigated using a single sputum sample from each participant. The Xpress assay detected SARS-CoV-2 in a similar proportion of cases to routine PCR (17.7% vs. 18.3%). There was substantial agreement between routine PCR and Xpress, suggesting that the swab capture method is reliable for COVID-19 diagnosis. MTBC was detected in 3% of participants and 1% of participants demonstrated dual detection of MTBC and SARS-CoV-2.

Participant recruitment coincided with SA’s fourth and fifth COVID-19 waves, when ~99% of sequenced specimens were identified as the Omicron variant of SARS-CoV-2 [21,22]. Other than cough and headache, which accounted for the majority of symptoms reported, common symptoms included sore throat, fatigue, nasal congestion/runny nose and self-reported fever/chills, each accounting for ≥35% of symptoms, which is consistent with the most common Omicron symptoms reported at that time [23]. Most participants seeking health care did so within days of symptom onset. Since diabetes is among the five key drivers of the global TB epidemic [3], this prompted the assessment of participant glucose levels. Although various factors can influence random glucose levels, 15 participants exhibited elevated glucose levels, which may increase their risk of developing TB.

There were 37 participants in whom SARS-CoV-2 was detected by Xpress but not by routine PCR. The findings by Marais et al. (2021) [24] indicate that SARS-CoV-2 detection in the early stages of infection (0–3 days) is more reliable in oral samples than in nasal samples, particularly for the Omicron variant. Since 43% of participants presented to the HCF within three days of symptom onset, this may explain the detection of the virus in sputum but not in NP specimens. These findings highlight the potential advantage of testing oral specimens in individuals presenting to healthcare facilities shortly after symptom onset. Another factor for consideration is the *S*-gene target failure observed with the TaqPath™ COVID-19 assay during the study period, which likely contributed to reduced assay sensitivity [25]. Notably, this assay was most used for routine PCR testing in Gauteng. This study focused on dual testing for TB and COVID-19; screening for other common seasonal pathogens, such as influenza, was not performed.

For the 35 participants in whom SARS-CoV-2 was detected by routine PCR but not by Xpress, one possible explanation is a known issue with the Xpress assay sensitivity due to delayed PCR hybridization due to a 1% drop in *E*-gene coverage [25].

As per the NHLS algorithm, specimens with Ct values close to the cut-off and meeting certain criteria were treated with caution and reported as ‘inconclusive’ rather than positive. This classification applied to 8% of study participants. Although these tests were not repeated in this study, the swab capture method allows for repeat testing if sputum is still available.

Of the 35 participants with MTBC detected, 15 were diagnosed during the fourth and fifth COVID-19 waves. During these peaks, most HCFs only referred patients for further diagnostics if they tested negative for SARS-CoV-2 and returned to facility if still unwell. Given their COVID-19 symptoms, these participants likely would not have been tested for TB and would have remained undiagnosed had they not been enrolled in this study. A notable portion of the TB-diseased population belonged to high-risk-for-TB groups, emphasizing the need for targeted interventions and effective management strategies for these individuals. Additionally, 7/35 (20%) were found to be RIF-resistant and although the sample size is small, this is higher than the reported estimated SA RIF-resistance rate of up to 4.3% [26].

It has been suggested that individuals with COVID-19 and concurrent TB face an increased likelihood of hospitalization, extended recovery times and a higher risk of premature death than those without TB [27]. For the ten participants who were found to be TB/SARS-CoV-2 co-infected in this study, it is likely that the risk of hospitalization and mortality was low due to the reduced clinical severity of the Omicron variant [28]. For symptomatic participants, 75% sought care within 10 days of symptom onset, which translates to the earlier diagnosis and management of both diseases. Ninety percent of co-infected participants were unvaccinated, which potentially increased their risk of contracting COVID-19. None had prior TB, suggesting SARS-CoV-2 may have triggered latent TB to progress to active disease or accelerated its progression, as reported in previous studies [11,29]. However, since both diseases were diagnosed simultaneously, the possibility also exists that these individuals first contracted TB, remained undiagnosed due to disrupted TB services [30] and, later, contracted COVID-19.

Initiatives such as TUTT, which were already being investigated pre-COVID-19, demonstrated that the under-diagnosis of TB is a reality. Active case-finding strategies are, therefore, critical. A similar initiative to TUTT, called “Tuberculosis Neighborhood Expanded Testing” or TB NET, [31] was launched by Médecins Sans Frontières and the City of Cape Town. This initiative screened households near index TB cases by requesting individuals to self-collect and drop off sputum at dedicated community points for TB testing. Despite challenges like household refusal to participate due to stigma and a low return rate of sputum jars (151/1400), the program achieved a 7.9% TB positivity rate among those tested.

The availability of multi-disease testing platforms, such as the GeneXpert system used in this study, is an important consideration for integrating diagnostic services. The WHO identified multi-disease testing as a key strategy in 2018 to simplify infectious disease diagnosis and aid in pandemic response [32]. While bi-disease testing has been implemented for HIV/TB [33] and COVID-19/TB [18], previous studies have not utilized a single specimen for both diseases. Our study is among the first to evaluate the feasibility of bi-disease testing for TB and COVID-19 from a single sputum sample. Although COVID-19 cases have declined (WHO COVID-19 dashboard; https://covid19.who.int, accessed 30 January 2025), this research highlights the value of bi-disease testing, particularly during pandemics when resources are diverted from routine programs. The implementation of integrated diagnostic strategies requires investment in specimen collection methods, health care settings (community-based vs. facility-based) and suitable diagnostic tools. However, such approaches could significantly enhance case detection and contribute to ending the TB epidemic. The use of a single sputum specimen for dual testing presents a practical, patient-centered solution that improves diagnostic efficiency, reduces the burden on healthcare systems and supports active case-finding efforts in high-TB-burden settings.

## 5. Conclusions

The study findings underscore a substantial identification of TB and rifampicin-resistant TB that would have been missed without a bi-disease testing approach. The swab capture method was also shown to be reliable for SARS-CoV-2 detection. Additionally, this approach demonstrates the feasibility of utilizing a single specimen on an analyzer with multiplexing capabilities. The implementation of such an innovative approach should be taken into consideration, not only for patient management but also for future pandemic preparedness.

## Figures and Tables

**Table 1:** Participant characteristics, by site and overall.

Characteristic	All *(n* = 1032)	CCHC (*n* = 506)	MCHC (*n* = 526)
*Demographics*			
Age, Mean (range), Years	41 (18–84)	39 (18–78)	44 (18–84)
Male sex, n (%)	459 (44.5)	190 (37.5)	269 (51.1)
*HIV-related information*			
HIV-positive, n (%)	248 (24.0)	102 (20.2)	146 (27.8)
HIV-negative, n (%)	744 (72.1)	371 (73.3)	373 (70.9)
Unknown, *n* (%)	40 (3.8)	33 (6.5)	7 (1.3)
Receiving ARV therapy, n/total (%)	227/248 (91.5)	90/102 (88.2)	137/146 (93.8)
Abnormal Random glucose levels, n/total (%)	15/1032 (1.5)	9/506 (1.7)	6/526 (1.1)
*Co-morbidities*			
Hypertension, n (%)	166 (16.1)	73 (14.4)	93 (17.7)
Diabetes, n (%)	48 (4.7)	27 (5.3)	21 (4.0)
Heart disease, n (%)	12 (1.2)	9 (1.8)	3 (0.6)
Other chronic lung diseases, n (%)	8 (0.8)	3 (0.6)	5 (0.9)
Cancer, n (%)	7 (0.7)	2 (0.4)	5 (0.9)
*TB History*			
Previously diagnosed with TB, n (%)	87 (8.4)	34 (6.7)	53 (10.1)
Contact of person diagnosed with TB, n (%)	30 (2.9)	21 (4.2)	9 (1.7)
*Clinical signs and symptoms at presentation*			
Symptom duration (days), Average (range)	5.2 (1–62 days)	3.9 (1–15 days)	6.5 (1–62 days)
Cough, n/total (%)	881/935 (94.2)	468/482 (96.5)	413/453 (91.2)
Headache, n/total (%)	680/935 (72.7)	405/482 (84.0)	275/453 (60.7)
Sore throat, n/total (%)	645/935 (68.9)	377/482 (78.2)	268/453 (59.2)
Fatigue, n/total (%)	515/935 (55.1)	240/482 (49.8)	275/453 (60.7)
Nasal congestion/runny nose, n/total (%)	424/935 (45.3)	268/482 (55.6)	156/453 (34.4)
Self-reported fever/chills, n/total (%)	328/935 (35.1)	242/482 (50.2)	86/453 (18.9)
Muscle pain, n/total (%)	264/935 (28.2)	82/482 (17.0)	183/453 (40.4)
Nights sweats, n/total (%)	255/935 (27.3)	145/482 (30.1)	110/453 (24.3)
Ageusia, n/total (%)	222/935 (23.7)	149/482 (30.9)	73/453 (16.1)
Loss of appetite, n/total (%)	206/935 (22.0)	134/482 (27.8)	72/453 (15.9)
Shortness of breath, n/total (%)	192/935 (20.5)	158/482 (32.8)	34/453 (7.5)
Unexplained weight loss, n/total (%)	173/935 (18.5)	87/482 (18.0)	86/453 (19.0)
Anosmia, n/total (%)	134/935 (14.3)	93/482 (19.3)	41/453 (9.1)
Abdominal pains, n/total (%)	104/935 (11.1)	52/482 (10.8)	52/453 (11.5)
Other ^1^, n/total (%)	101/935 (10.8)	58/482 (12.0)	43/453 (9.5)
GIT symptoms, n/total (%)	59/935 (6.3)	37/482 (7.7)	22/453 (4.9)
*COVID-19 vaccination status*			
Vaccinated, n/total (%)	563/1008 (55.9)	299/490 (61.0)	264/518 (50.9)
Asymptomatic, n/total (%)	97/1032 (9.4)	24/506 (4.7)	73/526 (13.9)

^1^ Other symptoms reported include chest pain, dizziness, stomach cramps, sweating and tonsillitis. CCHC, Chiawelo Community Health Centre; MCHC, Mofolo Community Health Centre; ARV, antiretroviral; TB, tuberculosis; GIT, gastro-intestinal tract; ARV, antiretroviral.

**Table 2:** Characteristics of participants with positive test results for TB and/or SARS-CoV-2 (routine PCR or Xpert Xpress swab capture).

Variable	TB Xpert MTB/RIF Ultra (*n* = 35)	Routine PCR for SARS-CoV-2 (*n* = 189)	Xpert Xpress for SARS-CoV-2 (*n* = 183)	TB/COVID Co-Infected (*n* = 10)
*Demographics*				
Age, Mean (range), Years	37 (20–74)	42 (18–74)	41 (18–74)	37 (22–52)
Male sex, n (%)	22 (62.8)	62 (32.8)	66 (36.1)	4 (40.0)
*HIV-related information*				
HIV-positive, n (%)	10 (28.6)	37 (19.6)	39 (21.3)	5 (50.0)
HIV-negative, n (%)	24 (68.6)	135 (71.4)	127 (69.4)	5 (50.0)
Unknown, n (%)	1 (2.9)	33 (17.5)	17 (9.3)	0 (0)
*Co-morbidities*				
Other chronic lung diseases, n (%)	1 (2.9)	2 (1.1)	2 (1.1)	1 (10.0)
Hypertension, n (%)	1 (2.9)	33 (17.5)	29 (15.9)	1 (10.0)
Heart disease, n (%)	0 (0)	6 (3.2)	2 (1.1)	0 (0)
Diabetes, n (%)	1 (2.9)	11 (5.8)	7 (3.8)	0 (0)
Cancer, n (%)	0 (0)	1 (0.5)	2 (1.1)	0 (0)
*Clinical signs and symptoms at presentation*				
Symptom duration (days), Average (range)	10.8 (3–60)	4.6 (1–38)	4.7 (1–38)	9.6 (3–21)
Cough, n/total (%)	31/34 (91.2)	165/181 (91.2)	155/171 (90.6)	8/10 (80.0)
Headache, n/total (%)	23/34 (67.6)	145/181 (80.1)	134/171 (78.3)	5/8 (62.5)
Sore throat, n/total (%)	23/34 (67.6)	133/181 (73.4)	128/171 (74.9)	7/8 (87.5)
Unexplained weight loss, n/total (%)	21/34 (61.2)	39/181 (21.5)	36/171 (21.1)	8/10 (80.0)
Nights sweats, n/total (%)	21/34 (61.2)	51/181 (28.2)	51/171 (29.8)	8/10 (80.0)
Loss of appetite, n/total (%)	20/34 (58.8)	45/181 (24.9)	42/171 (24.6)	6/10 (60.0)
Fatigue, n/total (%)	20/34 (58.8)	114/181 (62.9)	106/171 (62.0)	5/8 (62.5)
Nasal congestion/runny nose, n/total (%)	13/34 (38.2)	82/181 (45.3)	82/171 (48.0)	2/8 (25.0)
Muscle pain, n/total (%)	12/34 (35.3)	63/181 (35.2)	63/171 (36.8)	3/8 (37.5)
Ageusia, n/total (%)	11/34 (32.4)	47/181 (26.0)	45/171 (26.3)	2/8 (25.0)
Self-reported fever/chills, n/total (%)	7/34 (20.6)	73/181 (40.3)	71/171 (41.5)	1/8 (12.5)
Shortness of breath, n/total (%)	4/34 (11.8)	35/181 (19.3)	35/171 (20.5)	0/8 (0.0)
Anosmia, n/total (%)	4/34 (11.8)	27/181 (15.0)	27/171 (15.8)	1/8 (12.5)
Abdominal pains, n/total (%)	4/34 (11.8))	35/181 (19.3)	33/171 (19.3)	0/8 (0.0)
GIT symptoms, n/total (%)	4/34 (11.8)	8/181 (4.4)	8/171 (4.7)	1/8 (12.5)
Other ^1^, n/total (%)	2/34 (5.9)	11/181 (6.1)	15/171 (8.8)	0/8 (0.0)
Asymptomatic, n/total (%)	1/35 (2.9)	8/189 (4.2)	12/183 (6.6)	0/0 (0)

^1^ Other symptoms reported include chest pain, dizziness, stomach cramps, sweating and tonsillitis. TB, tuberculosis; PCR, polymerase chain reaction; GIT, gastro-intestinal tract; ARV, antiretrovirals.

**Table 3:** Comparison of routine SARS-CoV-2 results with Xpert Xpress swab capture results.

		Xpert Xpress Result			
Routine SARS-CoV-2 result		Positive	Negative	Total	Percent agreement *n/N* (%)
	Positive	146	35	181	PPA, 146/181 (81%)
	Negative	37	723	760	NPA, 723/760 (95%)
	Total	183	758	941	OPA, 869/941 (92%)

PPA, positive percent agreement; NPA, negative percent agreement; OPA, overall percent agreement. Xpert Xpress SARS-CoV-2 was used to detect SARS-CoV-2 on sputum swabs. Routine SARS-CoV-2 testing was performed on nasopharyngeal swabs.

## Data Availability

The raw data supporting the conclusions of this article will be made available by the authors on request.

## Supplementary Materials

The following supporting information can be downloaded at https://www.mdpi.com/article/10.3390/diagnostics15060720/s1; Table S1: Symptoms reported by participants.

## Abbreviations

The following abbreviations are used in this manuscript:

TB	tuberculosis
MTBC	*Mycobacterium tuberculosis* complex
Xpress	Xpert Xpress SARS-CoV-2
COVID-19	Coronavirus Disease 2019
TUTT	Targeted Universal Testing for TB
PLHIV	people living with HIV
HCFs	health care facilities
W4SS	WHO-recommended four-symptom screen
WHO	World Health Organization
mWRDs	WHO-approved rapid diagnostic tests
NP	nasopharyngeal
PHCH	primary health care clinics
PCR	polymerase chain reaction
TS	tongue swab
ARV	antiretroviral
